# TROP2 Expression in Sebaceous and Sweat Gland Carcinoma

**DOI:** 10.3390/jcm11030607

**Published:** 2022-01-25

**Authors:** Takamichi Ito, Hiroki Hashimoto, Yuka Tanaka, Keiko Tanegashima, Maho Murata, Toshio Ichiki, Takeshi Iwasaki, Yoshinao Oda, Yumiko Kaku-Ito

**Affiliations:** 1 Department of Dermatology, Graduate School of Medical Sciences, Kyushu University, Fukuoka 812-8582, Japan; h-hashi@dermatol.med.kyushu-u.ac.jp (H.H.); yukat53@med.kyushu-u.ac.jp (Y.T.); mpsyp388@yahoo.co.jp (K.T.); muratama@dermatol.med.kyushu-u.ac.jp (M.M.); itoshio@dermatol.med.kyushu-u.ac.jp (T.I.); kyumiko@dermatol.med.kyushu-u.ac.jp (Y.K.-I.); 2 Department of Anatomic Pathology, Graduate School of Medical Sciences, Kyushu University, Fukuoka 812-8582, Japan; iwasaki.takeshi.666@m.kyushu-u.ac.jp (T.I.); oda@surgpath.med.kyushu-u.ac.jp (Y.O.)

**Keywords:** trophoblast cell surface antigen 2 (TROP2), skin cancer, sebaceous carcinoma, sweat gland carcinoma, sacituzumab govitecan, targeted therapy, appendageal tumor, nectin cell adhesion molecule 4 (NECTIN4), adnexal carcinoma, malignant tumors of apocrine and eccrine differentiation

## Abstract

Sebaceous carcinoma and sweat gland carcinoma (malignant tumors with apocrine and eccrine differentiation) are rare malignant skin adnexal tumors that differentiate toward sebaceous gland and eccrine and apocrine glands, respectively. Owing to the rarity of these carcinomas, standard treatments for advanced disease have not been established. Because the prognosis of patients with systemic metastasis is poor, a new treatment for these diseases is eagerly desired. Trophoblast cell surface antigen 2 (TROP2) and sacituzumab govitecan, an antibody–drug conjugate of TROP2, have attracted attention in the treatment of various solid tumors. In the current study, we immunohistochemically investigated TROP2 expression in 14 sebaceous carcinoma and 18 sweat gland carcinoma samples and found strong and relatively homogeneous TROP2 staining in both cancer types. The mean Histoscore, a semi-quantitative scoring ranging from 0 (negative) to 300, was 265.5 in sebaceous carcinoma and 260.0 in sweat gland carcinoma. These observations directly suggest that both sebaceous carcinoma and sweat gland carcinoma could be potentially treated with TROP2-targeted antibody–drug conjugates such as sacituzumab govitecan.

## 1. Introduction

Sebaceous carcinoma is a malignant tumor consisting of basaloid or squamous atypical cells with sebaceous differentiation. Sebaceous carcinoma has been classified into two types (ocular and extraocular). The former type occurs on the eyelids and originates from the Meibomian glands and glands of Zeis. The latter type most commonly arises in the head and neck, but it can also occur in the anogenital area and other locations. Histopathologically, sebaceous carcinoma is composed of irregular lobules consisting of many undifferentiated cells and distinct sebaceous cells with foamy cytoplasm. These cells are atypical, displaying nuclear and nucleolar pleomorphism [1].

Sweat gland carcinomas (malignant tumors with apocrine and eccrine differentiation) comprise a heterogeneous group of carcinomas that are divided into 17 subgroups in the latest (4th) version of the World Health Organization (WHO) classification of skin tumors [2]. Owing to the rarity of sebaceous carcinoma and sweat gland carcinoma, no standard treatment for advanced disease has been established, and the prognosis of patients with systemic metastasis is poor. A new treatment for these diseases is eagerly desired.

Trophoblast cell surface antigen 2 (TROP2) is a surface glycoprotein originally identified in human placental trophoblasts [3,4,5]. TROP2 is highly expressed in various cancers such as pancreatic cancer [6], gastric cancer [7], lung cancer [3], colorectal cancer [8,9,10], and extramammary Paget’s disease [11]. Because TROP2 is involved in various malignant tumor processes including cancer proliferation, migration, invasion, and metastasis and TROP2 overexpression is associated with worse patient survival in several solid malignant tumors, TROP2 has attracted attention as a potential target for cancer therapy [9,12,13,14,15,16,17,18,19,20]. Recently, an antibody–drug conjugate (ADC) targeting TROP2 was revealed to have clinical benefits in clinical trials of lung, urothelial, breast, and other miscellaneous epithelial cancers [21,22,23,24,25].

We previously reported TROP2 expression in normal skin and skin appendages [11] and detected strong TROP2 expression in sebaceous and sweat glands. These findings prompted us to further investigate TROP2 expression in sebaceous carcinoma and sweat gland carcinoma. In the current study, we analyzed TROP2 expression in both malignancies and examined whether TROP2 could be a therapeutic target for these malignant tumors.

## 2. Materials and Methods

### 2.1. Ethics Statement

This study was conducted in accordance with the concepts enshrined in the Declaration of Helsinki, and approved by the Kyushu University Institutional Ethics Committee (approval ID: 30-363, approved on 27 November 2018). Written informed consent was obtained from the patients prior to their inclusion in the study.

### 2.2. Patients

We retrieved data from 14 patients with sebaceous carcinoma (14 samples) and from 13 patients with sweat gland carcinoma (18 samples) who were treated at the Department of Dermatology, Kyushu University (Fukuoka, Japan) between December 2008 and December 2018. At least three experienced dermatopathologists confirmed the diagnosis. To confirm tumor differentiation, an immunohistochemistry panel was used when necessary. The diagnosis was made in accordance with the latest version of the WHO classification of skin tumors [2]. The clinical and demographic data of all patients were collected from patients’ files and analyzed.

### 2.3. Immunohistochemistry

Immunohistochemical staining was performed as reported previously with slight modification [26,27,28]. Surgically obtained samples were fixed in 10% buffered formalin for 24 h and embedded in paraffin. The formalin-fixed paraffin-embedded tissues were cut into 4 μm-thick sections. TROP2 antigen was retrieved using Heat Processor Solution pH 9 (Nichirei Biosciences, Tokyo, Japan) via heating at 100 °C for 40 min. The sections were incubated with rabbit anti-human TROP2 (1:1000, ab214488; Abcam, Cambridge, UK) for 90 min at room temperature followed by incubation with N-Histofine Simple Stain MAX-PO MULTI (724152; Nichirei Biosciences) for 30 min at room temperature. Immunoreactions were detected using 3,3′-diaminobenzidine tetrahydrochloride (725191; Nichirei Biosciences) as a chromogen (exposure for 10 min), and specimens were counterstained using hematoxylin.

### 2.4. Evaluation of TROP2 Immunohistochemical Staining

The immunohistochemical results were evaluated by a semi-quantitative approach using the Histoscore (H-score) [29]. The intensity scores were 0 (no staining), 1+ (weak positivity), 2+ (moderate positivity), or 3+ (strong positivity). The epidermis was used as an internal control, and its score was 3+. The H-score of TROP2 was calculated as the percentage of positive cells (0–100%, either cytoplasmic or membranous staining) multiplied by the staining intensity (0–3+) The H-score, ranging from 0 to 300, was calculated by counting tumor cells in three random high-power fields (×200). For samples with both membranous and cytoplasmic staining, we recorded the stronger intensity of the staining. Two independent dermatologists (T.I. and Y.K.-I.) who were blinded to the clinical information assessed the sections. Images were taken using an ECLIPSE 80i microscope (Nikon, Tokyo, Japan). All statistical graphs were created using GraphPad Prism version 6.0 (GraphPad Software, San Diego, CA, USA).

## 3. Results

### 3.1. Patients and Tumors

**Figure 1:**
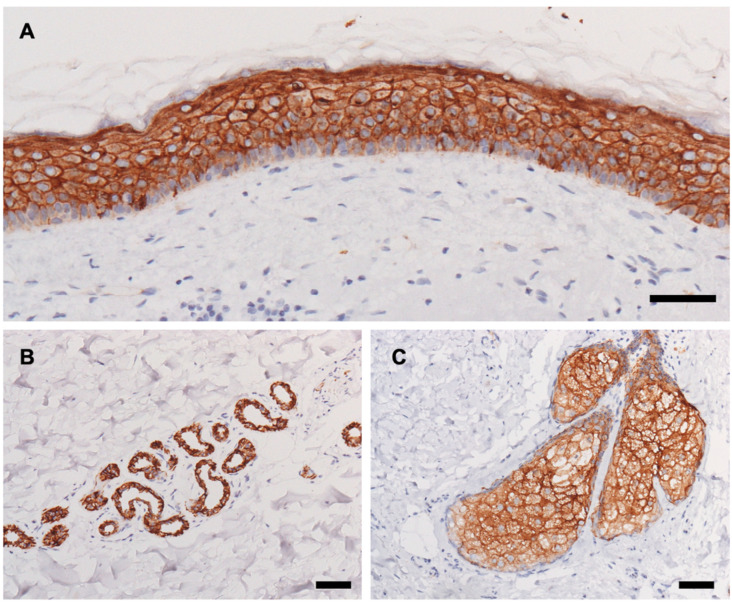
(**A**–**C**) Representative histopathological images of trophoblast cell surface antigen 2 (TROP2) staining in normal skin and skin appendages. (**A**) Epidermis. (**B**) Eccrine gland. (**C**) Sebaceous gland. Bars indicate 100 μm.

We collected 14 sebaceous carcinoma samples from 14 patients and 18 sweat gland carcinoma samples from 13 patients. The demographic data of patients with sebaceous carcinoma are presented in Table 1. Table 2 presents the demographic data of patients with sweat gland carcinoma. In addition to the 13 primary tumors, five metastatic tumors from three patients were examined. Representative image of normal skin epidermis and skin appendages are shown in Figure 1.

### 3.2. TROP2 Expression in Sebaceous Carcinoma

**Figure 2:**
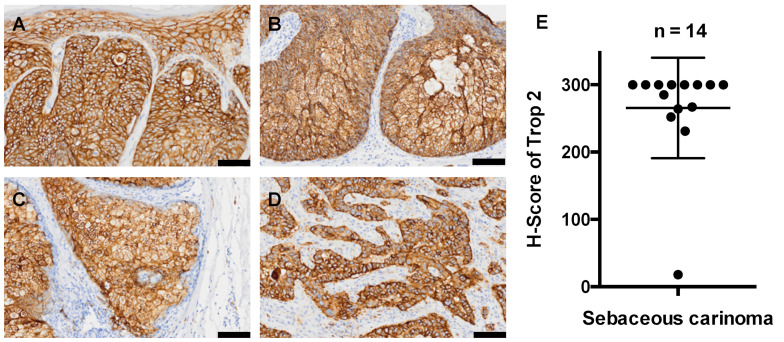
Histopathological images of trophoblast cell surface antigen 2 (TROP2) staining in sebaceous carcinoma. (**A**–**D**) Positive TROP2 signals were found on the membranes and in the cytoplasm of tumor cells. (**E**) The H-scores of TROP2 in all 14 sebaceous carcinoma samples. Bars indicate 100 μm.

Representative histopathological images of TROP2 are presented in Figure 2. Positive signals are indicated in brown. Most sebaceous carcinomas exhibited strong and homogeneous staining for TROP2 mainly in the cell membrane and partly in the cytoplasm (Figure 2A–D). The H-score, a semi-quantitative score (range, 0–300) obtained by multiplying the intensity score (range, 0 (negative staining) to +3 (strong staining)) by the proportion score (percentage of positive cells), was also interesting, as all but one tumor had high (>150) H-scores (Figure 2E). The mean and median H-scores were 265.5 and 300, respectively (Figure 2E).

### 3.3. TROP2 Expression in Sweat Gland Carcinoma

**Figure 3:**
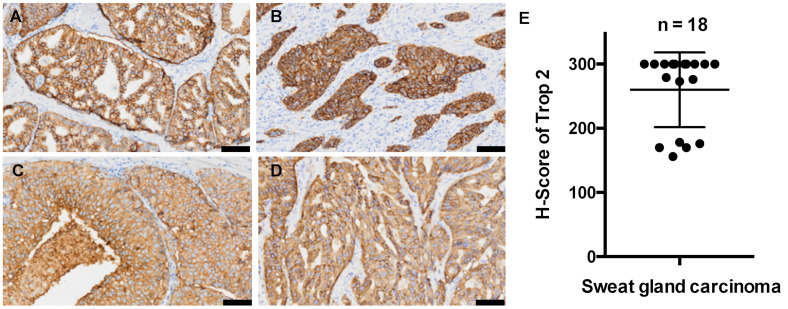
Histopathological images of trophoblast cell surface antigen 2 (TROP2) staining in sweat gland carcinoma. (**A**,**B**) Strongly positive staining (3+) in primary lesions. (**C**,**D**) Strongly positive staining (3+) in lymph node metastases. Positive TROP2 signals were found on the membranes and in the cytoplasm of tumor cells. (**E**) The H-scores of TROP2 in all 18 sweat gland carcinoma samples. Bars indicate 100 μm.

Representative images of TROP2 staining are presented in Figure 3. Similarly as sebaceous carcinoma, most sweat gland carcinoma samples displayed strong TROP2 expression in the membrane and moderate expression in the cytoplasm (Figure 3A–D) regardless of the tumor subtypes, and the mean and median H-scores were 260.0 and 300, respectively (Figure 3E). There was no obvious difference in the TROP2 expression between primary and metastatic tumors.

## 4. Discussion

Cutaneous adnexal tumors comprise a large and heterogeneous group of neoplasms that differentiate toward one or more skin appendages or recapitulate events occurring during embryo development. They include tumors with predominant apocrine, eccrine, follicular, sebaceous, or multilineage differentiation. Malignant adnexal tumors are less common than their benign counterparts [1,2]. Owing to the rarity of adnexal carcinoma, the best treatment strategy has not been established, particularly for metastatic lesions. ADCs, which consist of a monoclonal antibody linked to a cytotoxic drug, represent a new class of therapeutic drug designed for targeted therapy. Upon binding to a cell surface antigen, ADCs are internalized by tumor cells and processed by the endolysosomal system. The linker is then cleaved, and the cytotoxic drug is released into the cytoplasm, in which it induces cell apoptosis via its cytotoxic activity [30]. Although the ideal indications of ADCs are hematological malignancies in which lineage-specific antigens are expressed, ADCs targeting solid tumors have rigorously been explored in recent years. Several ADCs, such as trastuzumab emtansine (anti-HER2 antibody linked to the microtubule inhibitor maytansine), trastuzumab deruxtecan (anti-HER2 antibody linked to the topoisomerase inhibitor deruxtecan), enfortumab vedotin (anti-NECTIN4 antibody linked to the microtubule inhibitor monomethyl auristatin E), and sacituzumab govitecan (anti-TROP2 antibody linked to the topoisomerase inhibitor SN-38), are currently approved by the US Food and Drug Administration (FDA) for the treatment of breast cancer and urothelial cancer. Given the concept of ADCs, which facilitate intratumoral drug delivery via specific tumor cell surface antigens, various solid tumors could be targets for ADC if they express ADC-related antigens (e.g., HER2, NECTIN4, TROP2) [24,25,30,31,32,33,34]. In the current study, we found strong and relatively homogeneous TROP2 expression in the membrane in sebaceous carcinoma and sweat gland carcinoma, which directly suggests that the tumors are potential targets of TROP2-targeted ADCs (e.g., sacituzumab govitecan). TROP2 expression was extremely high in both sebaceous carcinoma and sweat gland carcinoma tissues, with mean H-scores of 265.5 and 260.0, respectively. More than half of sebaceous carcinoma and sweat gland carcinoma samples had a maximal H-score of 300.

The first-line treatment for localized sebaceous carcinoma and sweat gland carcinoma is complete surgical removal, which leads to a relatively good prognosis [35,36,37,38,39]. However, the survival of patients with metastatic lesions is dismal because of the lack of effective treatments. Although little guidance is available for the systemic treatment of advanced sebaceous carcinoma or sweat gland carcinoma, individual case reports and small case series suggest the use of anthracycline-, taxane-, or platinum-based regimens [35,36]. Recent studies suggested the potential use of agents targeting retinoic acid receptor-β, androgen receptor, mammalian target of rapamycin, or epidermal growth factor receptor; however, these drugs are not widely used, and their clinical benefits are largely unknown [35,36]. A new treatment strategy is required for unresectable sebaceous carcinoma and sweat gland carcinoma.

TROP2 has recently attracted attention as a potential target for anticancer therapy. It is involved in a variety of cell signaling pathways [40], and high TROP2 expression is associated with metastasis through its regulation of epithelial-to-mesenchymal transition and linked to the dismal prognosis of cancer [19,20,31,41]. Blockade of TROP2 using specific antibodies produced potential anticancer activities in head and neck squamous cell carcinoma [42] and pancreatic cancer [43]. Suppression of TROP2 by curcumin, a natural product, inhibited proliferation and motility in bladder cancer cells [44]. These results suggest that TROP2 inhibition could be an effective anticancer therapy. Another interesting strategy associated with TROP2 is ADC therapy. Recently, sacituzumab govitecan has received FDA approval for the treatment of triple-negative breast cancer, lung cancer and urothelial carcinoma [30]. Sacituzumab govitecan is a TROP2-targeted ADC that consists of a fully humanized IgG1 anti-TROP2 antibody and SN-38, an active metabolite of irinotecan. Upon antibody binding to the cell surface antigen TROP2, the linked SN-38 is released into the tumor microenvironment, leading to the death of nearby tumor cells via the bystander effect [30,45]. For breast cancer, the significant prolongation of progression-free and overall survival was validated in a phase III trial (NCT02574455) [24]. In addition, anticancer efficacy for a broad range of solid tumors and acceptable tolerability were demonstrated in a phase I/II basket trial (NCT01631552).

In conclusion, we observed TROP2 overexpression in sebaceous and sweat gland carcinomas. Most tumor cells exhibited high TROP2 expression, suggesting that TROP2-targeted therapies could be new treatment options for unresectable sebaceous carcinoma and sweat gland carcinoma.

## Figures and Tables

**Table 1:** Demographic data of 14 patients with sebaceous carcinoma.

Parameters		n (%)
Age, Years	Mean	73.7
	Median	75
	Range	47–95
Sex	Male	5 (35.7)
	Female	9 (64.3)
Primary tumor site	Head and neck	12 (85.7)
	Others	2 (14.3)
Primary tumor size	≤2 cm	9 (64.3)
	2–4 cm	5 (35.7)
	>4 cm	0 (0.0)
TNM stage at diagnosis	I–II	13 (92.9)
	III–IV	1 (7.1)

**Table 2:** Demographic data of 13 patients with sweat gland carcinoma.

Parameters		n (%)
Age, Years	Mean	67.1
	Median	64
	Range	46–89
Sex	Male	9 (69.2)
	Female	4 (30.8)
Tumor subtype	Apocrine carcinoma	6 (46.2)
	Porocarcinoma	3 (23.1)
	Others	4 (30.8)
Primary tumor site	Extremities	5 (38.5)
	Axilla	5 (38.5)
	Head and neck	2 (15.3)
	Trunk	1 (7.7)
Primary tumor size	≤2 cm	4 (30.8)
	2–4 cm	6 (46.2)
	>4 cm	3 (23.1)
TNM stage at diagnosis	I–II	4 (30.8)
	III–IV	9 (69.2)

## Data Availability

The data presented in this study are available in the main text.
